# Fresh Embryo Transfer Cycle Characteristics and Outcomes Following In Vitro Fertilization via Intracytoplasmic Sperm Injection Among Patients With and Without COVID-19 Vaccination

**DOI:** 10.1001/jamanetworkopen.2022.8625

**Published:** 2022-04-22

**Authors:** Emily Jacobs, Karen Summers, Amy Sparks, Rachel Mejia

**Affiliations:** 1 Division of Reproductive Endocrinology and Infertility, Department of Obstetrics and Gynecology, University of Iowa Hospital and Clinics, Iowa City

## Abstract

This cohort study examines the association of COVID-19 vaccination status and in vitro fertilization (IVF)-fresh embryo transfer cycle stimulation characteristics and outcomes.

## Introduction

Women of reproductive age have been at the forefront of COVID-19 vaccine hesitancy, citing concerns about the vaccine’s effect on future fertility, current pregnancy, and breastfeeding (among others).^1^ As of February 2022, only 57% of pregnant patients were fully vaccinated against COVID-19 prior to becoming pregnant, a rate that lags that of the general population.^2^ To date, current literature surrounding COVID-19 vaccination and potential associations with infertility have been performed mainly in frozen embryo transfer cycles or in vitro fertilization cycles (IVF) using intracytoplasmic sperm injection (ICSI), both of which do not occur in in vivo conception.^3,4^ The aim of this study was to investigate the association of COVID-19 vaccination status with IVF-fresh embryo transfer cycle stimulation characteristics and clinical outcomes.

## Methods

The University of Iowa institutional review board approved this retrospective cohort study. Patients undergoing IVF-fresh embryo transfer cycles at a single academic institution from December 14, 2020, to September 30, 2021, were included. All patients gave written informed consent to be included in our IVF institutional research database. Cycle characteristics and clinical outcomes were compared between COVID-19 vaccinated and unvaccinated patients. Vaccination status was determined by accessing immunization records in the electronic medical record for each patient. Bivariate analysis was performed using *t* tests, Mann-Whitney *U* tests, and χ^2^ tests. Generalized estimating equations were used to control for multiple cycles per patient and odds ratios were calculated adjusting for age and body mass index (BMI). In addition, subanalysis was performed for (1) exclusion of day 5 morula transfers and (2) standard insemination–only cycles. Additional methods are described in the eAppendix of the Supplement. Statistical analysis was performed using SPSS version 27 (IBM Corp), and a 2-sided *P *< .05 was considered statistically significant.

## Results

Study demographics and clinical characteristics are shown in Table 1; 142 patients were vaccinated against COVID-19 and 138 patients were unvaccinated. The majority of patients were young, nulliparous, and overweight. As seen in Table 1, the 2 groups were similar at baseline. In the vaccinated group, 127 patients (89.7%) were fully vaccinated and 15 patients (10.6%) were partially vaccinated. The mean (SD) time from last vaccination to oocyte retrieval was 93 (65) days. There was no difference in ovarian reserve (mean [SD] antral follicle count for vaccinated group: 23 [13] vs unvaccinated group: 24 [15]; *P* = .42) or ovarian response (mean [SD] days of gonadotropin stimulation for vaccinated group: 9.8 [1.6] vs unvaccinated group: 9.6 [1.4]) (Table 1). The mean (SD) number of oocytes retrieved (vaccinated group: 14 [8] vs unvaccinated group: 15 [9]) and mean (SD) number of useable embryos produced (vaccinated group: 4 [3] vs unvaccinated group: 4 [3]) were also similar between groups (Table 2). Vaccinated patients had higher mean (SD) fertilization rates than unvaccinated patients (77.45% [41.45%] vs 68.66% [20.51%]; *P* = .03). In addition, after controlling for factors that can influence IVF success (age and BMI), there were no significant differences in ongoing clinical pregnancy rate (adjusted odds ratio [aOR], 0.79; 95% CI, 0.48-1.29) and miscarriage rate (aOR, 2.15; 95% CI, 0.62-7.47) in vaccinated vs unvaccinated patients. A subanalysis excluding day 5 morula transfers found no difference in ongoing clinical pregnancy rate (aOR, 0.82; 95% CI, 0.49-1.36) and miscarriage rate (aOR, 1.28; 95% CI, 0.32-5.07). Lastly, when comparing standard insemination-only cycles, there was also no difference in ongoing clinical pregnancy (aOR, 0.94; 95% CI, 0.47-1.87) or miscarriage rate (aOR, 4.09; 95% CI, 0.35-47.74).

**Table 1.** Study Demographics and Clinical Characteristics

Characteristic	Vaccinated (n = 142)	Unvaccinated (n = 138)
Demographics		
Age, mean (SD), y	34 (4)	33 (4)
BMI, mean (SD)	28.08 (7.43)	29.28 (7.25)
Weight, mean (SD), kg	80.42 (21.36)	79.58 (21.31)
Gravida, median (IQR)	0 (0-1)	1 (0-2)
Para, median (IQR)	0 (0-1)	0 (0-1)
Vaccination characteristics, No. (%)		
Doses of vaccine		
Partially vaccinated	15 (10.6)	NA
Fully vaccinated	127 (89.7)	NA
Vaccine name, No. (%)		
mRNA-1273 (Moderna)	70 (49.3)	NA
BNT162b2 (Pfizer-BioNTech)	65 (45.8)	NA
Ad26.COV2.S (Janssen)	7 (4.9)	NA
Time between last vaccine dose and retrieval, mean (SD), d	93 (65)	NA
Cycle characteristics		
Cycle No., median (IQR)	1 (1-2)	1 (1-1)
Protocol, No (%)		
Antagonist	13 (9.2)	7 (5.1)
Dual trigger	67 (47.2)	64 (46.4)
Long agonist	32 (22.5)	33 (23.9)
Microdose flare	17 (12.0)	15 (10.9)
Estrogen prime	13 (9.2)	19 (13.8)
Diagnosis, No. (%)		
AMA	19 (13.4)	12 (8.7)
DOR	20 (14.1)	20 (14.5)
Anovulation	13 (9.2)	16 (11.6)
Male Factor	42 (29.6)	45 (32.6)
Endometriosis	13 (9.2)	13 (9.4)
Tubal factor	17 (12.0)	20 (14.5)
RPL	6 (4.2)	1 (0.7)
Uterine/cervical	1 (0.7)	1 (0.7)
Unexplained	34 (23.9)	33 (23.9)
Antral follicle count, mean (SD)	23 (13)	24 (15)
Insemination method, No. (%)		
Regular	66 (46.5)	74 (53.6)
ICSI	76 (53.5)	64 (46.4)
Duration of stimulation, mean (SD)	9.8 (1.6)	9.6 (1.4)
No. transferred, No. (%)		
SET	20 (14.1)	20 (14.5)
eSET	93 (65.5)	91 (65.9)
DET	29 (20.4)	27 (19.6)

Abbreviations: AMA, advanced maternal age; BMI, body mass index, calculated as weight in kilograms divided by height in meters squared; DET, double embryo transfer; DOR, diminished ovarian reserve; eSET, elective single embryo transfer (at least 1 additional embryo available to cryopreserve); ICSI, intracytoplasmic sperm injection; RPL, recurrent pregnancy loss; SET, single embryo transfer (no embryos available to cryopreserve).

**Table 2.** Cycle Characteristics and Clinical Outcomes in COVID-19 Vaccinated and Unvaccinated Patients

Cycle characteristics	Mean (SD)		Mean difference (95% CI)	*P* value
	Vaccinated (n = 142)	Unvaccinated (n = 138)		
No. of oocytes retrieved	14 (8)	15 (9)	–1 (–3 to 1)	.33
No. of oocytes inseminated	12 (7)	13 (8)	–1 (–3 to 1)	.31
No. of 2PN	9 (8)	8 (5)	1 (–1 to 2)	.34
Fertilization rate				
No. of 2PN/No. of oocytes inseminated	77.45 (41.54)	68.86 (20.51)	8.78 (1.02 to 16.56)	.03
Blastulation rate				
No. of blastocysts/No. of 2PN (%)	44.37 (25.55)	46.79 (26.54)	2.42 (–3.71 to 8.55)	.44
No. of blastocysts per retrieval	4 (3)	4 (3)	0 (–1 to 1)	.83
No. of embryos cryopreserved	3 (3)	3 (3)	0 (–1 to 1)	.73
**Clinical outcomes**	**Vaccinated**	**Unvaccinated**	**OR (95% CI)** ^a^	**aOR (95% CI)** ^a^
Full sample, No.	142	138	NA	NA
Ongoing clinical pregnancy, No. (%)	65 (45.8)	74 (53.6)	0.73 (0.45 to 1.18)	0.79 (0.48 to 1.29)
Miscarriage, No. (%)	8/73 (11.0)	4/78 (5.1)	2.28 (0.66 to 7.87)	2.15 (0.62 to 7.47)
Subsample excluding day 5 morula transfers, No.	130	125	NA	NA
Ongoing clinical pregnancy	65 (50.0)	71 (56.8)	0.76 (0.46 to 1.25)	0.82 (0.49 to 1.36)
Miscarriage	5/70 (7.1)	4/75 (5.3)	1.37 (0.35 to 5.26)	1.28 (0.32 to 5.07)
Subsample with only standard insemination cycles, No.	66	74	NA	NA
Ongoing clinical pregnancy	34 (51.5)	41 (55.4)	0.86 (0.44 to 1.67)	0.94 (0.47 to 1.87)
Miscarriage	3/37 (8.1)	1/42 (2.4)	3.62 (0.36 to 36.39)	4.09 (0.35 to 47.74)

Abbreviations: aOR, odds ratios adjusted for age and body mass index; NA, not applicable; OR, unadjusted odds ratio; 2PN, 2 pronuclear.

^a^ Generalized estimating equations used to control for multiple cycles per patient.

## Discussion

To our knowledge, this is one of the first studies to evaluate the association of COVID-19 vaccination status with IVF-fresh embryo transfer cycles (including a high proportion of standard insemination cycles). We found no evidence to suggest that COVID-19 vaccination negatively affects cycle stimulation characteristics, embryological variables, or clinical outcomes in IVF. Current and emerging scientific evidence continues to support that COVID-19 vaccination is safe and effective and has no impact on fertility. The results of this study can be used to provide reassuring data to patients planning on pregnancy considering COVID-19 vaccination.

## Notes

Supplement. **eAppendix.** Online Only Supplemental Material
eReferences
