# Supplementary material for: Fresh Embryo Transfer Cycle Characteristics and Outcomes Following In Vitro Fertilization via Intracytoplasmic Sperm Injection Among Patients With and Without COVID-19 Vaccination
Source: JAMA Netw Open. 2022 Apr 22;5(4):e228625. doi: 10.1001/jamanetworkopen.2022.8625 (PMC9034396; doi:10.1001/jamanetworkopen.2022.8625)
Supplement: Supplement. — eAppendix. Online Only Supplemental Material eReferences [file jamanetwopen-e228625-s001.pdf]

Supplemental Online Content

Jacobs E, Summers K, Sparks A, Mejia R. Fresh embryo transfer cycle characteristics and outcomes following in vitro fertilization via intracytoplasmic sperm injection among patients with and without COVID-19 vaccination. *JAMA Netw Open.* 2022;5(4):e228625. doi:10.1001/jamanetworkopen.2022.8625

eAppendix. Online Only Supplemental Material
eReferences

This supplemental material has been provided by the authors to give readers additional information about their work.

© 2022 Jacobs E et al. *JAMA Network Open.*

eAppendix. Online Only Supplemental Material

The University of Iowa institutional review board approved this retrospective study (IRB 20170974). All patients were consented prior to the start of their IVF cycle for their information to be stored and used in future research at our institution. Exclusion criteria included donor oocytes and gestational carrier cycles. We followed STROBE reporting guidelines for cohort studies. In total, 298 IVF– fresh embryo transfer cycles were performed during the study period and 18 were excluded (17 donor oocyte cycles and 1 gestational carrier cycle). Fully vaccinated patients were defined as having received two doses of an mRNA vaccine (Pfizer or Moderna) or one dose of an adenovirus vaccine (Johnson & Johnson). Partially vaccinated patients were defined as having received one dose of an mRNA vaccine. We did not report the vaccination status of the male partner due to previously published studies that have shown no difference in sperm parameters before and after COVID-19 vaccination[1]. Ongoing clinical pregnancy was defined as ultrasonographic visualization of a gestational sac with a fetal pole and detectable fetal heart rate. Miscarriage was defined as loss of a pregnancy after ultrasonographic identification of a fetal heart rate. In our sensitivity power analysis, we assumed the ongoing clinical pregnancy rates in the unvaccinated group would be similar to our clinic's live birth rates. With alpha set at 0.05, our sample of 138 cycles in unvaccinated patients and 142 cycles in vaccinated patients would have 80% power to detect a 17% absolute difference in ongoing clinical pregnancy rates between the two groups.

eReferences

1. Gonzalez DC, Nassau DE, Khodamoradi K, et al. Sperm Parameters Before and After COVID-19 mRNA Vaccination. *JAMA.* 2021;326(3):273–274. doi:10.1001/jama.2021.9976

© 2022 Jacobs E et al. *JAMA Network Open.*
